# Evidence That Cardiac Pulse Strains Retinal Vessels in and near the Optic Disc During Ocular Ductions

**DOI:** 10.3390/bioengineering13070725

**Published:** 2026-06-24

**Authors:** Emanuil Parunakian, Atharva Shetye, Veronika Yehezkeli, Somaye Jafari, Joseph L. Demer

**Affiliations:** 1 David Geffen School of Medicine, University of California Los Angeles, Los Angeles, CA 90095, USA; eparunakian@mednet.ucla.edu; 2 Department of Ophthalmology, Stein Eye Institute, University of California, Los Angeles, CA 90095, USA; atharvashetye@ucla.edu (A.S.); sjafari@mednet.ucla.edu (S.J.); 3 Department of Mechanical Engineering, University of California, Los Angeles, CA 90095, USA; 4 Department of Neurology, University of California, Los Angeles, CA 90095, USA; 5 Bioengineering Department, University of California, Los Angeles, CA 90095, USA; 6 Neuroscience Interdepartmental Program, University of California, Los Angeles, CA 90095, USA

**Keywords:** cardiac pulse, ocular biomechanics, optic disc, scanning laser ophthalmoscopy, strain

## Abstract

Ocular ductions deform the optic disc and peripapillary blood vessels, and deformations can be interpreted as mechanical strain. We used confocal scanning laser ophthalmoscopy (cSLO) to map strain in disc and peripapillary retinal vessels associated with the cardiac pulse and determine if such strain is influenced by gaze direction. Sets of 13 infrared cSLO images were obtained sequentially for each eye using a Heidelberg Spectralis scanner in cinematic mode over a 3 sec interval in adults. Imaging was repeated in central, and horizontally (30° adduction/abduction) and vertically eccentric gazes (10° supraduction/infraduction). Retinal vessels, optic disc, and fovea were segmented using custom-trained, deep learning-based models. Frame to frame vascular displacements were automatically determined using optical flow analysis, allowing computation of equivalent strain. A total of 25 eyes of 13 subjects of mean age 39 ± 18 (standard deviation, range: 25 to 81) years were included. Average equivalent strain over 3 sec ranging from 0.27% to 0.36% exceeded the 0.16% noise threshold across all gazes and regions, indicating measurable pulse-induced deformation. After adjustment for age and axial length, pulsatile maximum and minimum strain were influenced slightly by gaze direction, maximally for supraduction, whereas mean strain did not vary significantly with gaze. The cardiac pulse induces measurable deformation of retinal vessels that can be quantified as equivalent strain in the image plane using optical flow-derived displacement fields. However, the interaction of pulse strain with gaze direction is unlikely to be a significant confound for investigations of strains associated with eye movements.

## 1. Introduction

Advances in imaging have enabled direct visualization and quantification of minute deformations in the optic disc and peripapillary regions that are important for clarifying ocular physiology and its disorders. Various imaging modalities, including scanning laser Doppler flowmetry and time-resolved optical coherence tomography (OCT), have demonstrated fluctuations in blood flow velocity and retinal vessel diameter with the cardiac pulse [1,2]. Pulsatile deformations of optic disc and peripapillary vessels arise from blood flow into the retinal and choroidal vasculature and accompanying intraocular pressure (IOP) fluctuations during each cardiac cycle, linking vascular dynamics with tissue biomechanics and potentially with pathophysiology [2,3,4,5,6,7].

The introduction of OCT has also allowed detailed analysis of the biomechanics of optic disc structure [8]. Phase-sensitive and other specialized OCT methods have since demonstrated pulsatile deformations of a few microns in the optic disc during the cardiac cycle [9,10]. However, OCT acquisition speed and its limited field of view constrain the ability to capture rapid or spatially extensive vascular motion. Angiographic OCT (OCT-A) allows for the visualization of intravascular flow regions, but similar to structural OCT, the images are static and the low acquisition rate prohibits analysis of flow dynamics and pulsatile behavior [11].

Optic disc and peripapillary vessel deformation are demonstrable during eye movements in both healthy and pathological eyes, refs. [12,13,14,15] highlighting the presence of mechanical loading by the optic nerve on the posterior pole. Quantifying vessel strain caused by eye movements may provide important insights into how these biomechanical forces are distributed within ocular tissues and how repetitive mechanical stress may contribute to the development and progression of ocular pathologies [12,16]. Multiple prior studies used static confocal scanning laser ophthalmoscopy (cSLO) to assess gaze-induced deformation of retinal vessels within and around the optic disc [12,16]. In contrast, cSLO video sequences capture dynamic changes in vessel morphology associated with the cardiac pulse, raising the possibility that pulsatile deformation may have a confounding influence on measurements that are attributed to gaze-induced strain. However, the contribution of the cardiac pulse to retinal vessel strain and its potential confounding interaction with gaze direction have not been investigated. To address this gap, we developed and applied a novel analysis framework that combines machine learning-based vessel segmentation with deep learning-based optical flow analysis to quantify cardiac pulse-induced apparent strain in retinal vessels from infrared cSLO video sequences. By examining vessel deformation across horizontal and vertical eccentric gaze positions, this study aims to determine the contribution of the ocular pulse to optic disc and peripapillary retinal vessel strain during ocular ductions.

## 2. Materials and Methods

This study was approved by the University of California, Los Angeles Institutional Review Board and conducted in accordance with the Declaration of Helsinki and the U.S. Health Insurance Portability and Accountability Act (HIPAA). Volunteers free of ocular or systemic pathology that would restrict eye movements gave written informed consent before participation. Axial length of each eye was measured with IOLMaster 500 (Carl Zeiss Meditec AG, Jena, Germany).

**Figure 1:**
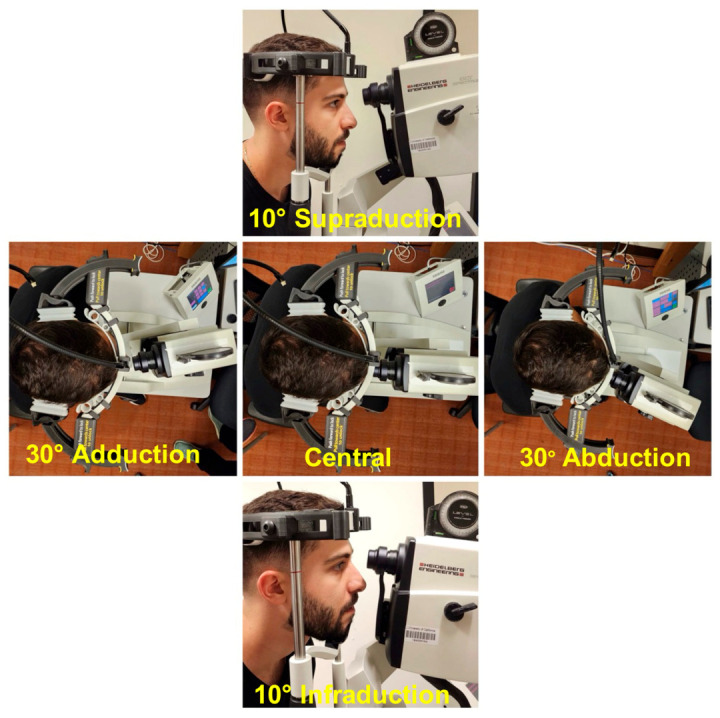
Images were acquired in central, horizontal, and vertical gaze directions.

To capture the effect over at least one cardiac cycle, three sec video clips (4.7 frames per sec) were recorded in infrared cSLO mode of the Heidelberg Spectralis scanner (Heidelberg Engineering Inc., Heidelberg, Germany). This instrument allows image acquisition speed to be traded against resolution. Images were acquired in a high-resolution (1536 × 1536 pixels) mode to optimize anatomical visualization, at the cost of modestly lower acquisition rate. Image acquisition was repeated for each subject in both eccentric and central gaze positions. Head position was stabilized using a custom-made head holder attached to the cSLO scanner. Imaging was performed during fixation of the scanner’s internal target, but with the scanner camera rotated on its gimbal to sequentially achieve central gaze, 30° ad- and abduction, and 10° supraduction and infraduction (Figure 1). All gaze positions accounted for offset of the internal fixation target of the scanner, as described previously [12,14]. Images were exported in “.tiff” format as sequences of 13 frames per gaze position.

Nonuniform illumination in the images was corrected prior to segmentation. Retinal vessels, optic disc, and fovea were segmented using separate supervised DeepLabV3+ models implemented in PyTorch 2.5.1. Ground truth masks for model training were manually annotated by three experienced ophthalmologists. The dataset consisted of 104 retinal cSLO images from 52 unique subjects and was adequate for robust model training. Because manual annotation is labor-intensive and requires careful expert review, the dataset size was limited to 104 images. Subject-level separation was performed prior to augmentation to prevent data leakage between training and validation sets, with 42 subjects assigned to the training set and 10 subjects assigned to the validation set. Data augmentation, including horizontal and vertical flipping, contrast and brightness adjustments, and noise addition, was then applied independently within each set to increase sample diversity while preserving dataset independence, resulting in approximately 5000 images for training and validation. All models were trained on 1536 × 1536 images using a CUDA-enabled NVIDIA RTX A6000 GPU with a batch size of 4. The models were optimized using the Adam optimizer with a learning rate of 1 × 10^−4^. Binary Cross-Entropy with Logits Loss (BCEWithLogitsLoss) was used during training, with class-balancing weights of 20, 30, and 90 applied for optic disc, retinal vessel, and fovea segmentation, respectively, to address class imbalance. Each model was trained for 25 epochs until convergence, and final model selection was based on validation performance.

**Figure 2:**
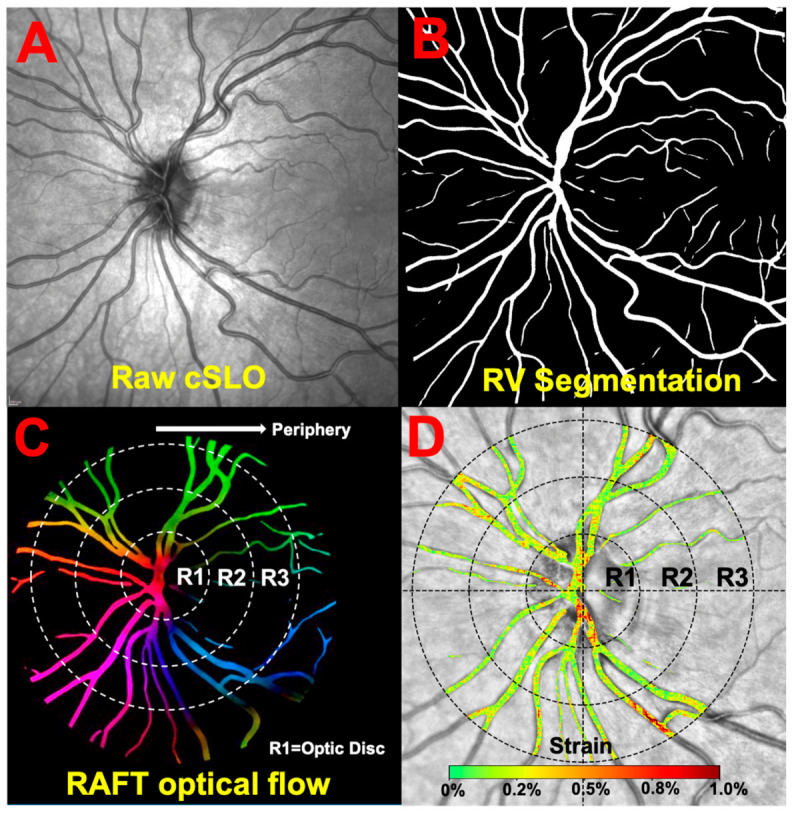
Image processing sequence during infraduction in a 37-year-old subject. (**A**). Raw confocal scanning laser ophthalmoscopy (cSLO) image. (**B**). Retinal vessel (RV) segmentation using DeeplabV3+. (**C**). RAFT optical flow to track vessel movement and region demarcation with colors from the Hue Saturation and Brightness (HSB) circle representing line of displacement, and brightness representing magnitude. Red represents rightward motion. Cyan represents leftward motion. Green/yellow represents downward motion. Magenta pink represents upward motion. (**D**). Heat map displaying strain. Region 1 (R1) = Optic disc; Region 2 (R2) = Peripapillary, Region (R3) = Peripheral.

Model performance was evaluated by computing the area under the receiver operating curve (AUROC), and the DICE similarity coefficient (F1-score) computed on a pixel-wise basis. AUROC quantifies the model’s ability to discriminate vessels from background pixels over the full range of thresholds, while DICE measures the spatial overlap between predicted and ground truth segmentations, balancing precision and recall. Even after illumination correction, the contrast of fine vessels remained highly sensitive to image brightness. Small vessels provide limited spatial information and are more susceptible to noise and motion artifacts, reducing their reliability for motion analysis. Therefore, retinal vessel segmentation emphasizes well-defined, high-contrast vessels. This approach reduced sensitivity to very fine vasculature but minimized false positive detections and improved robustness of motion estimation. For fovea segmentation, the model was trained on images containing both clearly defined and poorly visible or absent foveal features to improve generalizability. In the only two cases where the model failed to detect the fovea due to indistinct anatomical features in cSLO images, foveal location was annotated manually. The optic disc and fovea were used to compute the disc–foveal angle for torsion correction, while retinal vessel masks were used to quantify vessel motion across different gaze directions. The overall image processing pipeline is summarized in Figure 2.

The first frame that was free of artifact in each sequence was treated as the reference image, against which subsequent frames were compared. Sequential image acquisition had unknown relationship to the phase and frequency of the cardiac cycle. By selecting the first usable frame as the reference, a consistent baseline was established against which vessel shape changes induced by the cardiac pulse are quantified.

Scale Invariant Feature Transform (SIFT)-based registration was applied to align each subsequent frame to the reference frame, thereby correcting for global rigid body motion arising from eye or head movements during acquisition [17]. Feature correspondences were detected using standard SIFT descriptors, and a global transformation (translation and rotation) was estimated and applied to each frame prior to optical flow computation.

Displacements in the retinal vessels induced by the pulse were estimated using a Recurrent All-pairs Field Transforms (RAFT) deep learning optical flow algorithm described by Teed and colleagues [18]. This pretrained RAFT model used a 4-layer correlation pyramid and a lookup radius of 4, enabling sensitivity to small and rapid displacements.

Applying 2D continuum mechanics, the Green–Lagrange strain tensor was computed for each frame relative to the reference. The Green–Lagrange strain tensor provides an objective Lagrangian measure of deformation that remains valid for both finite and small strain regimes. Because the measured displacement gradients were small, the quadratic terms were negligible, and the formulation approaches the infinitesimal strain tensor in the small-deformation limit [19,20]. Equivalent strain was then computed from the deviatoric strain components to provide a scalar measure of distortion independent of direction. Thus,

(1) $$E_{eq}=\frac{2}{3}\sqrt{\left(\frac{3}{2}\left(e_{xx}^{2}+e_{yy}^{2}\right)+3E_{xy}^{2}\right)}$$

where, $e_{xx}\;and\;e_{yy}\;$ are the deviatoric strain components in *X* and *Y* directions and $E_{xy}$ represents in-plane shear strain of the Green-Lagrange strain tensor.

Equivalent strains were visualized as time histograms. Analysis was performed for three annular regions centered at the optic disc center, each of one disc radius width (Figure 2). For visualization, strain heatmaps were generated for each frame, both for the full image and for vessel specific regions obtained by applying a retinal vessel mask of that frame.

**Figure 3:**
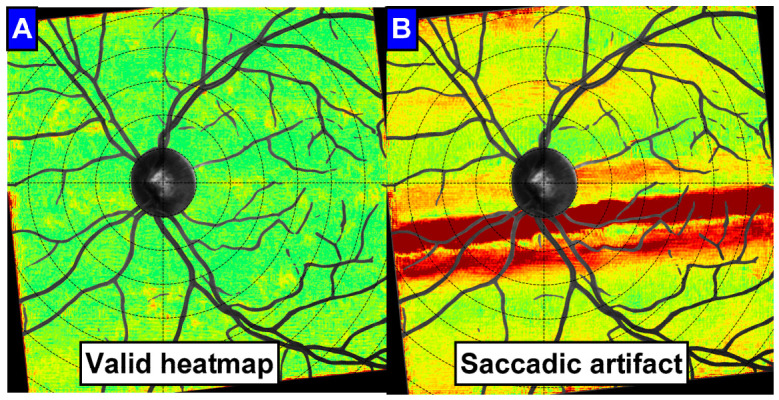
Strain heatmap during a stable image (**A**) versus an obviously corrupted image by a saccade during image acquisition that would have generated extreme apparent vascular strain necessitating exclusion from analysis (**B**).

Occasional motion artifacts due to saccades were identified by gross distortions of these strain heatmaps. Frames exhibiting striking linear horizontal bands correlating to the saccade were classified as artifactual, as these patterns are grossly inconsistent with localized pulsatile deformation (Figure 3). If the reference image itself were to have been compromised by a saccade, then all the subsequent stable images would have consistent linear pattern at the same location of the reference image saccade. In occasional cases where the reference image was corrupted by a saccade, the analysis was redone by excluding the compromised first frame and treating the next frame in the sequence as the reference. Since strain was computed based on motion estimates from RAFT optical flow, very high apparent strain values provided objective indication for rejection of corrupted image frames.

To characterize algorithmic noise, each frame was also compared with itself using the RAFT optical flow and strain computation pipeline. Ideally, this should yield zero strain, so any non-zero residual values represent experimental or computational noise. The mean and variance of these residual strain values across all frames was computed, providing both a measure of bias (mean) and precision (variance) of the algorithm.

The contribution of the cardiac pulse was automatically quantified during a 3 s interval that included at least one cardiac cycle by calculating the minimum and maximum equivalent strains. The difference between minimum and maximum equivalent strain reflected the vascular deformation induced by the cardiac pulse that would otherwise be within the noise floor due to limited movements between the frames. To compute the net strain due to cardiac pulse during the 3 s time frame, equivalent strain was averaged across the entire image sequence, which was defined as the mean strain.

To assess whether the observed strain signals exhibited temporal oscillatory behavior, frequency-domain analysis was performed on image sequences obtained in central gaze. This analysis was not intended to precisely quantify cardiac pulse rate but to evaluate oscillatory pattern in strain consistent with vascular pulsatility. A fast Fourier transform (FFT) was applied to strain time series derived from 13 frames in each image acquisition. Frame timestamps were approximated assuming uniform temporal spacing, derived from the total video duration and number of frames. In occasional cases where exclusion of one or more frames due to artifacts resulted in non-uniform temporal sampling, a Lomb–Scargle periodogram [21] modification of the FFT from the MATLAB R2024b signal processing toolkit. Because retained frames were timestamped and missing strain values due to corrupted frames were sparse, it was possible to estimate the dominant frequency from unevenly spaced data without interpolation. The Lomb–Scargle approach fits sinusoidal models across a range of frequencies and identifies one that best explains the observed variations. This allows robust frequency estimation despite missing data points. However, given the limited number of frames and short acquisition duration, frequency estimates were used to assess plausible consistency with expected heart rate rather than to derive precise pulse rates.

Statistical analysis was performed using IBM SPSS Statistics (version 31.0.0). Generalized estimating equations (GEEs) were used to evaluate associations between retinal strain parameters and gaze direction while accounting for repeated measurements obtained from both eyes of the same subject across multiple gaze positions. Separate GEE models were fitted for maximum strain, mean strain, and minimum strain. Eye and gaze direction were treated as repeated within-subject factors. Gaze direction was modeled as a categorical fixed effect using central gaze as the reference category, while age and axial length were included as covariates. Models assumed a normal probability distribution with identity link function and an exchangeable working correlation matrix. Robust standard errors were used for inference. Overall model effects were assessed using Type III Wald chi-square statistics. Estimated marginal means and corresponding 95% confidence intervals were calculated for each gaze direction after adjustment for age and axial length. Beta (β) in GEE represents the population-averaged regression coefficient. It quantifies the average change in the outcome variable associated with a one-unit increase in a predictor variable, while accounting for the correlation among repeated observations or clustered data within subjects.

## 3. Results

### 3.1. Subjects

This study included 25 eyes of 13 subjects (5 males 8 females), of mean age 39 ± 18 (standard deviation, SD, range: 25 to 81) years. Mean ocular axial length was 24.4 ± 1.3 mm (range: 22.3–26.9 mm).

### 3.2. Noise and Artifact Exclusion

Mean noise of the optical flow-based strain computation was consistent across all gaze directions at 0.16% ± 0.0001% equivalent strain, so that values below this are considered indistinguishable from computational noise.

The number of frames excluded for saccadic or artifactual images did not vary appreciably among gaze positions. Across all gazes, approximately 90% of eyes required exclusion of only 0–2 frames, while three frames were excluded in approximately 10% of cases.

### 3.3. Image Segmentation Accuracy

The model performed well, with performance metrics for segmentation summarized in Table 1.

### 3.4. Periodicity

**Figure 4:**
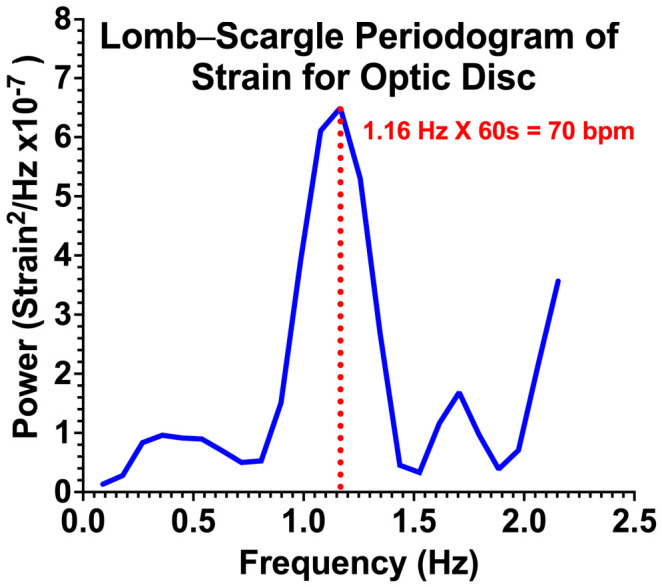
Lomb–Scargle periodogram spectral plot of pulse-induced strain in the optic disc (region 1) in central gaze for a representative 30-year-old subject.

**Figure 5:**
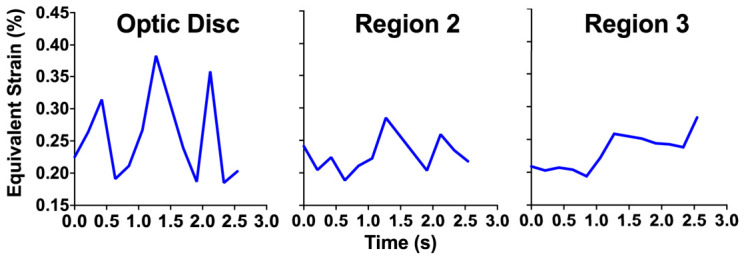
Equivalent pulsatile strain decrement with distance from the optic disc (region 1) through 3 for central gaze in representative 30-year-old subject. Region 1 (R1) = Optic disc; Region 2 (R2) = Peripapillary, Region (R3) = Peripheral.

For 25 eyes in central gaze, the dominant frequency of equivalent strain averaged 1.16 ± 0.32 Hz, corresponding to 70 beats per minute. This lies within the normal resting heart rate range of healthy adults (Figure 4). Periodicity was most evident within the optic disc (region 1). Equivalent strain amplitude decreased with distance from the disc in over 90% of eyes (Figure 5).

### 3.5. Pulse-Induced Strain

**Figure 6:**
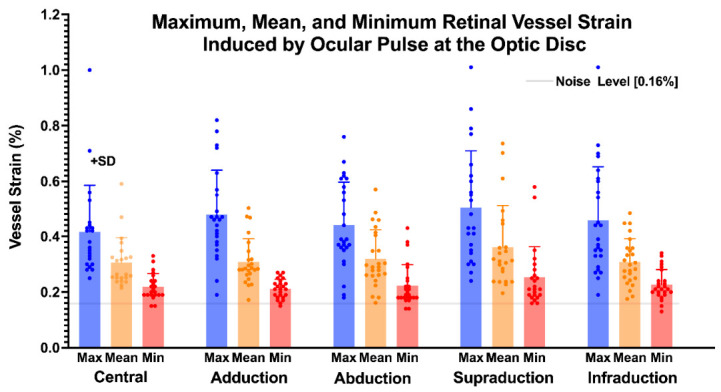
Minimum (red), mean (orange), and maximum (blue) vessel strain induced by ocular pulse in horizontal and vertical ductions at the optic disc. SD, standard deviation. Horizontal gray line represents repeatability noise level.

Maximum pulsatile retinal vessel strain was greatest in the disc and decreased progressively toward the periphery (regions 2–3). Average strain over 3 sec ranging from 0.27% to 0.36% exceeded the 0.16% noise threshold across all gazes and regions. In the optic disc (region 1), all average equivalent strains exceeded the 0.16% noise threshold (gray horizontal line, Figure 6), indicating that the computed strains are not attributed to the noise of the method but instead reflect physiologic changes in vessel structure.

Pulse-induced strain measurements across gaze positions are summarized in Table 2. Across all strain metrics, supraduction demonstrated the highest observed strain values, whereas central gaze and adduction generally demonstrated lower strain values.

Generalized estimating equation (GEE) analysis of pulsatile strain is summarized in Table 3. After adjustment for age and axial length, gaze direction demonstrated a significant effect on maximum strain (Wald χ^2^ = 10.398, *p* = 0.034) and minimum strain (Wald χ^2^ = 44.094, *p* < 0.001), whereas the effect of gaze on mean strain was not significant (Wald χ^2^ = 6.608, *p* = 0.158). Relative to central gaze, supraduction demonstrated significantly greater maximum strain (β = 0.079, *p* = 0.013), mean strain (β = 0.054, *p* = 0.020), and minimum strain (β = 0.035, *p* = 0.031). For no other gaze direction did pulse-induced strain by any metric differ significantly from that of central gaze.

Increasing age was significantly associated with higher maximum strain (β = 0.004, *p* = 0.002), mean strain (β = 0.003, *p* < 0.001), and minimum strain (β = 0.001, *p* = 0.003). Greater axial length was associated with lower maximum strain (β = −0.050, *p* = 0.034) and lower mean strain (β = −0.025, *p* = 0.034), whereas there was no significant association between axial length and minimum strain (*p* = 0.376).

Adjusted estimated marginal means are summarized in Supplementary Table S1. After adjustment for age and axial length, strains by all metrics were highest for supraduction.

## 4. Discussion

We here introduce a novel method for quantifying cardiac pulse-induced deformation in retinal vessels using infrared cSLO analyzed by deep learning-based image processing. The current study confirms and extends an early cSLO study that qualitatively described spontaneous venous pulsations of retinal vessels. However, early analysis was not automated, did not assess strain, and did not examine different gaze directions [22]. By integrating machine learning segmentation with optical flow-derived displacement analysis, we quantified vascular deformation patterns in retinal vessels that varied temporally in a manner consistent with the cardiac cycle. These deformation patterns are most plausibly associated with vascular transmission of the cardiac pulse.

Cardiac pulse-related retinal vessel deformation quantified as equivalent strain exceeded the computational noise threshold (0.16%) of our method in central and secondary gaze directions. The average cardiac pulse-induced equivalent strain (~0.3%) in retinal vessels at the optic disc is substantially lower than the ~2% equivalent strain reported at the optic disc in prior studies during eye rotations [12]. Strain was maximal within the optic disc (region 1), but decreased with distance from the disc. This topographic distribution aligns with prior physiological observations that the optic disc is the primary site of vascular pulsation transmission due to the entry and exit of the central retinal artery and vein [23,24]. These findings are consistent with a study using time-resolved structural OCT demonstrating diminishing pulsatility toward the periphery [25]. Under the present acquisition and analysis conditions, gaze direction significantly influenced maximum and minimum strains, but not mean strain. After adjustment for age and axial length, strains were greatest in supraduction, although the physiological significance of these differences remains uncertain given the modest sample size and limited temporal sampling. These vascular findings contrast with an earlier OCT-based study that identified larger pulsatile deformation of the whole optic disc in abduction than in the central gaze, in subjects with globe AL less than 25 mm [9]. The discrepancy likely reflects different phenomena: OCT captures structural deformation of neural and connective tissues, whereas our SLO-based approach quantifies vascular deformation in the image-plane derived from optical flow. It is unclear why maximum and mean strains were greater in eyes with shorter ALs, but the effect of AL was small. Cardiac pulse strain was greater in older than younger subjects. Deformation of retinal vessels reflects systemic hemodynamics such as blood pressure that clearly increase with age [26,27], as well as the tendency of local tissues to stiffen with age [28,29,30,31].

Other studies have employed various methods including Doppler OCT, laser speckle flowgraphy, and variable interscan time analysis of OCT angiography to visualize flow changes within ocular vessels [32,33,34]. One study used Doppler Fourier domain OCT to calculate average flow speeds but found no correlation with vessel diameter [35]. While Fourier domain OCT can estimate blood flow velocity, this technique is constrained by the need to accurately determine the Doppler angle. Laser speckle flowgraphy has demonstrated correlations with ocular circulation indices and optic disc structure [36], but laser speckle flowgraphy does not provide depth-resolved measurements or absolute measures of flow. Time-resolved dynamic structural OCT, a widely available clinical modality, can visualize depth- and time-resolved blood flow profiles in one or a few vessels at a time [25], but cannot generate a map of strain over a large area of the retina. These methods have all been focused on blood flow profiles and have not quantified the mechanical changes induced by the cardiac pulse that are described here.

The current study has some limitations. We did not perform simultaneous cardiac monitoring (e.g., electrocardiogram, pulse oximetry), which could directly confirm the cardiac origin of the observed oscillatory strains. We did not measure IOP, which might impact some of the observed strains. Our method does not differentiate between arteries and veins. Another limitation of this study is the temporal sampling as longer duration video acquisition made maintaining stable fixation for extended periods difficult for many subjects, thereby limiting sequential image acquisition to 3 s.

The consistency of the noise floor at 0.16% and low variance of 0.0001% strain across all gazes confirms the robustness of our optical flow-based computation, therefore reinforcing the validity of our method for subtle retinal deformations. These results align with similar optical flow applications in biomedical imaging that demonstrate subpixel precision for analysis of periodic motion [37]. By distinguishing cardiac pulse-induced strain from eye-movement-related strain, this study shows that vascular pulsatility would be expected to have minimal effect on imaging-based assessment of retinal vascular deformation associated with eye movements. However, application of this method for other purposes should consider the effects of hemodynamic factors.

## Figures and Tables

**Table 1:** Image segmentation model performance.

Feature	AUROC	F1/DICE Score
Retinal Vessels	0.97	0.82
Optic Disc	0.99	0.96
Fovea	0.97	0.88

**Table 2:** Pulse-induced retinal strain.

Gaze Position	Maximum Strain (%) (Mean ± SD)	Mean Strain (%) (Mean ± SD)	Minimum Strain (%) (Mean ± SD)
Abduction	0.44 ± 0.18	0.32 ± 0.11	0.22 ± 0.07
Adduction	0.48 ± 0.18	0.31 ± 0.11	0.21 ± 0.07
Central	0.42 ± 0.18	0.31 ± 0.11	0.22 ± 0.07
Infraduction	0.46 ± 0.18	0.31 ± 0.11	0.22 ± 0.07
Supraduction	0.50 ± 0.18	0.36 ± 0.11	0.25 ± 0.07

**Table 3:** Generalized estimating equation analysis of pulse-induced retinal strain differences from central gaze.

Outcome	Predictor	β	95% CI	Wald χ^2^	*p*-Value
Maximum Strain	Overall gaze effect	—	—	10.398	0.034
	Abduction vs. Central	0.013	−0.049 to 0.075	0.168	0.682
	Adduction vs. Central	0.054	−0.021 to 0.129	1.997	0.158
	Infraduction vs. Central	0.034	−0.043 to 0.111	0.751	0.386
	Supraduction vs. Central	0.079	0.017 to 0.141	6.200	0.013
	Age	0.004	0.002 to 0.007	9.600	0.002
	Axial Length	−0.050	−0.096 to −0.004	4.477	0.034
Mean Strain	Overall gaze effect	—	—	6.608	0.158
	Abduction vs. Central	0.007	−0.053 to 0.068	0.056	0.813
	Adduction vs. Central	−0.001	−0.039 to 0.037	0.001	0.972
	Infraduction vs. Central	−0.001	−0.035 to 0.032	0.007	0.933
	Supraduction vs. Central	0.054	0.009 to 0.100	5.427	0.020
	Age	0.003	0.001 to 0.004	14.953	<0.001
	Axial Length	−0.025	−0.048 to −0.002	4.505	0.034
Minimum Strain	Overall gaze effect	—	—	44.094	<0.001
	Abduction vs. Central	0.002	−0.034 to 0.037	0.008	0.930
	Adduction vs. Central	−0.009	−0.030 to 0.011	0.786	0.375
	Infraduction vs. Central	0.005	−0.016 to 0.026	0.219	0.640
	Supraduction vs. Central	0.035	0.003 to 0.068	4.679	0.031
	Age	0.001	0.000 to 0.002	8.645	0.003
	Axial Length	−0.005	−0.017 to 0.006	0.784	0.376

## Data Availability

In conformity with requirements of the funding agency the U.S. Public Health Service, National Institutes of Health, the anonymized raw numerical data for this study are provided for public access via Zenodo with the link 10.5281/zenodo.18357111. Being potentially individually identifiable, the cSLO images are not available for public access.

## Supplementary Materials

The following supporting information can be downloaded at https://www.mdpi.com/article/10.3390/bioengineering13070725/s1, Table S1: Marginal Mean Strains and Confidence Intervals for Retinal Vessel Strain After Adjustment for Age and Axial Length.

## Abbreviations

cSLO	confocal Scanning Laser Ophthalmoscopy
OCT	Optical Coherence Tomography
IOP	Intraocular Pressure
OCT-A	Angiographic Optical Coherence Tomography
HIPPA	Health Insurance Portability and Accountability Act
AUROC	Area Under the Receiver Operating Characteristic Curve
RV	Retinal Vessel
SIFT	Scale Invariant Feature Transform
RAFT	Recurrent All-pairs Field Transforms
FFT	Fast Fourier Transform
