# Supplementary material for: Evidence That Cardiac Pulse Strains Retinal Vessels in and near the Optic Disc During Ocular Ductions
Source: Bioengineering (Basel). 2026 Jun 24;13(7):725. doi: 10.3390/bioengineering13070725 (PMC13405817; doi:10.3390/bioengineering13070725)
Supplement: Supplementary file 1 [file bioengineering-13-00725-s001.zip › bioengineering-4313591-supplementary.pdf]

bioengineering

MDPI

Supplementary Table S1. Marginal mean strains and confidence intervals for retinal vessel strain after adjustment for age and axial length.

Outcome	Gaze Position	Adjusted Mean	95% CI
**Maximum Strain (%)**	Central	0.423	0.357–0.490
	Abduction	0.437	0.393–0.480
	Adduction	0.477	0.411–0.543
	Infraduction	0.457	0.382–0.533
	Supraduction	0.502	0.433–0.571
**Mean Strain (%)**	Central	0.308	0.271–0.346
	Abduction	0.316	0.284–0.347
	Adduction	0.308	0.278–0.337
	Infraduction	0.307	0.273–0.341
	Supraduction	0.362	0.310–0.414
**Minimum Strain (%)**	Central	0.219	0.197–0.241
	Abduction	0.220	0.198–0.243
	Adduction	0.209	0.195–0.224
	Infraduction	0.224	0.204–0.244
	Supraduction	0.254	0.218–0.291
